# Draft genome sequencing and *in vitro* activity data of the lytic vB_EcoM_AMO3598 bacteriophage effective against multi - drug resistant *E. coli*

**DOI:** 10.1016/j.dib.2025.111937

**Published:** 2025-08-05

**Authors:** Madina Alexyuk, Andrey Bogoyavlenskiy, Kuralay Akanova, Yergali Moldakhanov, Adolat Manakbayeva, Timur Kerimov, Vladimir Berezin, Pavel Alexyuk

**Affiliations:** Research and Production Center for Microbiology and Virology, 050010, Bogenbay batyr. Str., 105, Almaty, Kazakhstan

**Keywords:** Bacteriophage, *Escherichia coli*, Antibiotic resistance, Illumina sequencing, Genome annotation

## Abstract

One of the significant challenges of modern public health is the global spread of drug resistance among bacterial pathogens. According to WHO forecasts mortality from resistant bacterial infections by 2050 may reach up to 10 million cases per year. One of the ways to solve this problem is the mass introduction of phage therapy into medical practice, which requires the search, isolation, and study of lytic bacteriophages that can effectively fight resistant bacterial infections. In this study, we report on the genome of the lytic vB_EcoM_AMO3598 bacteriophage isolated from wastewater in Almaty city, targeting clinical, antibiotic-resistant *E. coli* strains. Bacteriophage vB_EcoM_AMO3598 possesses a double-stranded linear DNA genome of 145,425 bp in length containing 290 putative genes encoding proteins, including tRNA, and is a representative of the subfamily of *Stephanstirmvirinae*, genus *Justusliebigvirus*. The structure of the viral particles of phage vB_EcoM_AMO3598 has a myo-like shape with an icosahedral head and a contractile tail. The vB_EcoM_AMO3598 bacteriophage is known to be capable of lysing at least 4 clinical antibiotic-resistant *E. coli* strains. Up to 90% of viral particles are adsorbed within the first 4 minutes of phage infection. The latent period is 20 minutes, and the burst size is 218±28 phage particles per cell.

Specifications Table

**Table:** 

Subject	Health Sciences, Medical Sciences & Pharmacology
Specific subject area	Microbiology, Virology, Bacteriophage genomics.
Type of data	Table, Graph, Figure. Raw and processed sequencing data, genome annotation and phylogenetic analysis.
Data collection	Trypticasein Soy Broth (TSB, Condalab, Spain), Trypticasein Soy Agar (TSA, Condalab, Spain), and shaker-incubator INC 125 FS digital (IKA, Germany) were used for the growth of bacterial cultures. Phage lysates were purified from bacterial cells by sedimentation on a Centrifuge 5430R (Eppendorf, Germany) and filtration through SPHEROS Syringe filters, PES, 0.45 µm, sterile (LLG-Labware, Germany). Transmission electron microscopy was performed on an HT7800 (Hitachi, Japan). Phage DNA was extracted using PureLink Viral RNA/DNA mini kit, and WGS was performed on Illumina MiSeq sequencer with a paired-end library of a read length of 2 × 300 bp. The genome was assembled using the SPAdes 3.12.0 assembler. Predicted coding sequences (CDS) were annotated using PROKKA 1.7 (12) and manually edited. The coding regions of tRNAs were identified using tRNAscan-SE. PhageLeads together with the integrated ABRicate tool were used to check for the absence of lysogeny factors, antimicrobial resistance (AMR) genes and virulence genes. The VICTOR web service was used to generate a phylogenetic tree based on the classification of the complete genomes of prokaryotic viruses.
Data source location	Research and Production Center for Microbiology and Virology, Almaty, Kazakhstan. • City/Region: Almaty • Country: Kazakhstan • Latitude and longitude for collected samples: 43.24776402 N 76.94454847 E
Data accessibility	Direct URL to BioSample: https://www.ncbi.nlm.nih.gov/biosample/?term=SAMN47875066 Direct URL to BioProject: https://www.ncbi.nlm.nih.gov/bioproject/?term=PRJNA1249015 Direct URL to Whole Genome Data: https://www.ncbi.nlm.nih.gov/nuccore/2897486975 Direct URL to Raw Sequencing Data: https://www.ncbi.nlm.nih.gov/sra/?term=PRJNA1249015

## 1 Value of the Data

• Phage therapy is a promising method for combating antibiotic-resistant bacterial infections; however, to improve its effectiveness, a constant search and study of new lytic bacteriophages are required.
• The combination of phage genomics and *in vitro* tests enables the identification of potential biomarkers and the assessment of phage effectiveness as a therapeutic agent.
• The presented data show that the genome of the vB_EcoM_AMO3598 bacteriophage does not contain sequences encoding genes for antibiotic resistance, virulence and lysogeny.
• Data on the genes that allow vB_EcoM_AMO3598 bacteriophage to recognize and lyse host cells may be useful in modifying bacteriophages and developing new therapeutic agents against antibiotic-resistant bacterial infections.
• The obtained data are uploaded to the NCBI database and can be freely used by researchers developing alternative antimicrobial strategies, synthetic biology approaches or phage therapy methods aimed at combating antibiotic-resistant bacterial infections.

## 2 Background

Nosocomial infections are a global problem of modern healthcare. The threat posed by this problem is increasing annually due to the widespread antibiotic resistance among bacterial pathogens and the diminishing effectiveness of antibiotic therapy [1].

One of the causative agents of nosocomial infections is *E. coli*, mainly a commensal gram-negative bacterium that inhabits the gastrointestinal tract of warm-blooded animals, including humans. However, pathogenic forms of *E. coli* are also known to cause pneumonia, peritonitis, urinary tract infections, gastrointestinal tract infections, central nervous system infections, and soft tissue infections [2]. Patients with compromised immune systems are at high risk: nosocomial infections caused by antibiotic-resistant *E. coli* may result in acute bacteremia, sepsis, and potentially death [3].

The situation is further complicated by the ongoing decline in funding and research activity related to the development of new antibiotics. The continuation of this trend will eventually lead to the exhaustion of funds to fight resistant bacterial infections [4].

In such circumstances, interest in studying bacteriophages and phage therapy as an alternative or supplement to antibiotic therapy has significantly increased. A large number of examples of successful use of bacteriophages for the treatment of antibiotic-resistant bacterial infections are already known [5]. However, further work on isolating new lytic bacteriophages and their comprehensive study is needed to improve the efficacy of phage therapy and introduce it more widely into medical practice. This data article describes the whole-genome sequencing and *in vitro* activity data of the lytic bacteriophage vB_EcoM_AMO3598 isolated from wastewater of Almaty city.

## 3 Data Description

The studied bacteriophage was isolated from wastewater samples collected from wastewater treatment plants of Almaty, Kazakhstan located at the following geographical coordinates - N 43°24′14.1 “E 76°53′20.3″. The bacteriophage was isolated on the clinical, antibiotic-resistant strain *E. coli* 3598. The study of the spectrum of lytic activity of the isolated bacteriophage made it possible to establish that in addition to the host bacterium (*E. coli* 3598), this bacteriophage had the ability to completely inhibit the growth of 3 more clinical antibiotic-resistant strains of *E. coli* out of the 12 studied (Table 1).

**Table 1:** Spectrum of lytic activity of the vB_EcoM_AMO3598 bacteriophage and antibiotic resistance of clinical strains of *E. coli.*

Clinical *E. coli* strain	The level of lytic activity	Antibiotic resistance spectrum of clinical strains of E. coli**											
		AMP	AMC	TZP	CXM	CTX	CAZ	FEP	ETP	MEM	AMK	GEN	LEV
3598	+++*	R***	S	S	R	R	R	R	S	S	S	R	R
3640	—	R	S	S	R	R	R	R	S	S	S	S	R
3679	—	R	S	S	I	R	I	I	S	S	S	S	R
3692	—	R	R	R	R	R	R	R	S	S	S	S	R
3693	—	R	S	S	R	R	R	R	S	S	S	S	R
3701	+++	I	S	S	I	R	I	I	S	S	S	S	R
3706	+++	R	R	R	R	R	R	R	S	S	S	S	R
3819	+++	R	R	R	R	R	R	R	S	S	S	R	R
3826	—	R	S	S	R	R	R	R	S	S	S	S	R
3828	—	R	R	S	R	R	R	R	S	S	S	S	R
3849	—	R	R	S	R	R	R	R	S	S	I	R	R
3868	—	R	R	R	R	R	R	R	S	S	S	R	R

*+++ - extensive lysis zone without secondary growth of bacterial colonies, — - no lysis zone.

** sensitivity data were obtained on a bioMerieux^Ⓡ^ analyzer and provided together with *E. coli* strains.

*** R – resistance; I - intermediate; S – sensitive.

**Fig. 1:**
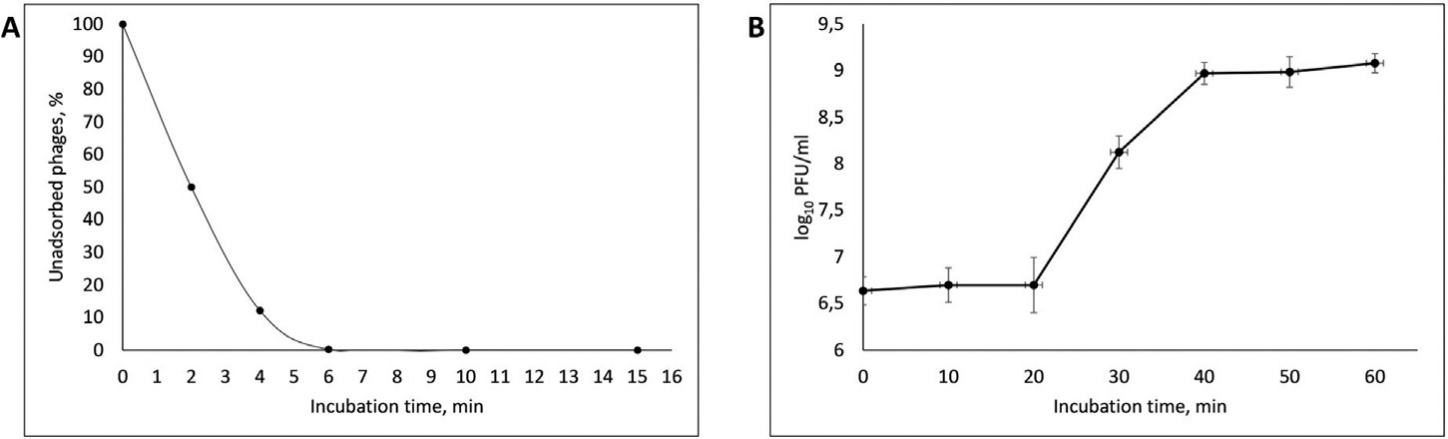
Adsorption rate (A) One-step growth curve (B) of the vB_EcoM_AMO3598 bacteriophage.

As a result of determining the adsorption rate, it was found that 4 min after the start of incubation, up to 90 % of the vB_EcoM_AMO3598 bacteriophage viral particles were adsorbed to the host bacteria (Fig. 1A). The one-step growth curve showed that the latent period of vB_EcoM_AMO3598 bacteriophage was 20 min, then a burst size period was recorded, it lasted from 20 to 40 min, after which the titer of viral particles reached a plateau (Fig. 1B). The burst size averaged 218±28 phage particles per cell.

**Fig. 2:**
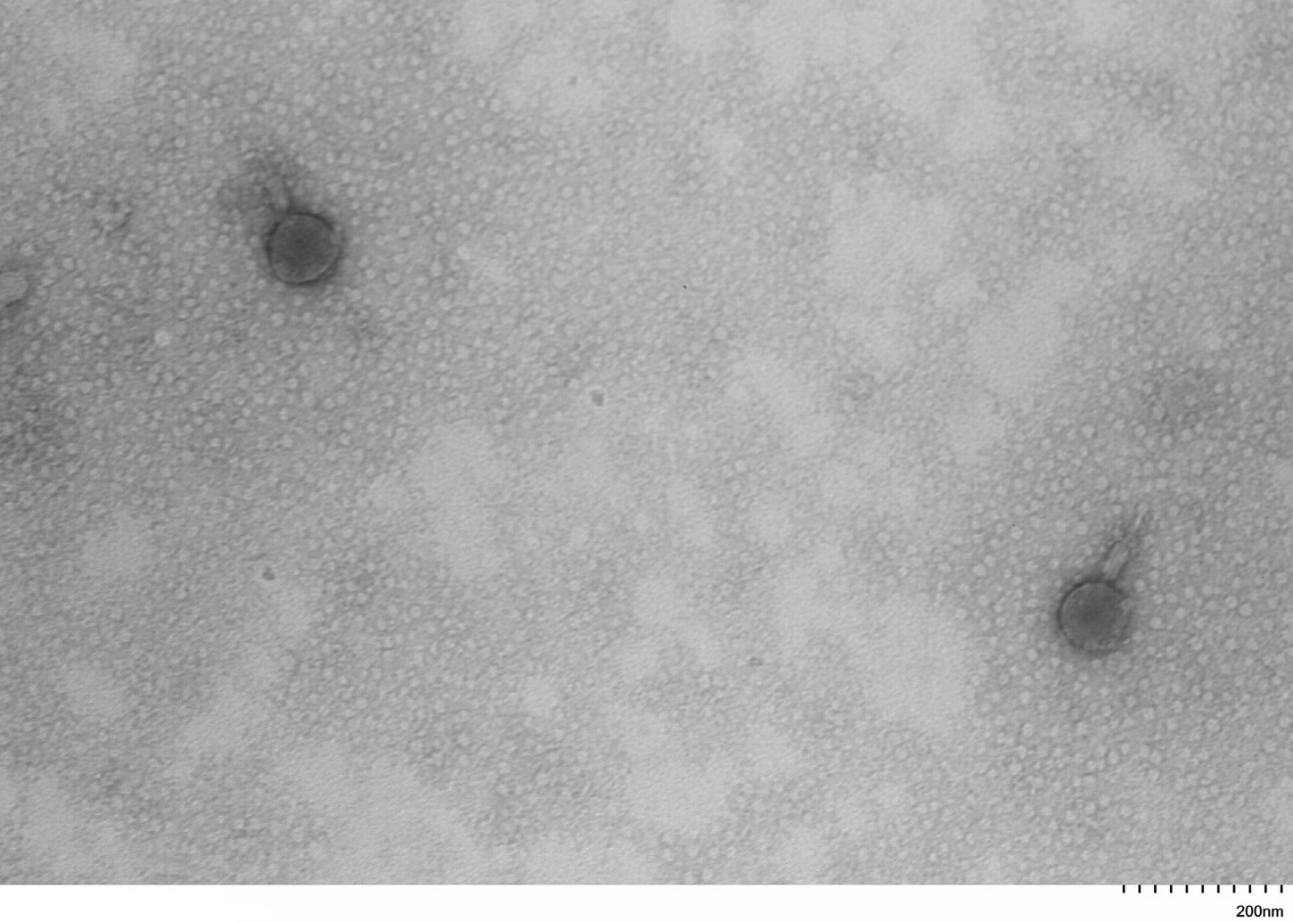
Transmission electron micrographs of the vB_EcoM_AMO3598 bacteriophage.

Transmission electron microscopy revealed that the studied virus belonged to the myoviruses group with an icosahedral head with a diameter averaging 100 nm and a contractile tail up to 100 nm long (Fig. 2).

The bacteriophage vB_EcoM_AMO3598 possesses a double-stranded linear DNA genome of 145,425 bp in length with a *G* + *C* content of 37.5 % (Table 2). The closest related phage is the Escherichia phage pEC-M2929–1AR.1 (OP177727) with an average nucleotide identity (ANI) of 98.8 %.

**Table 2:** Genome details of the vB_EcoM_AMO3598 bacteriophage.

Feature	vB_EcoM_AMO3598
Genome size, bp	145,425
ORF number	277
GC content	37.5 %
tRNAs	13
Morphology	Myovirus
Closest relative	Escherichia phage pEC-M2929–1AR.1 (OP177727)
Accession no.	PQ226818.1

**Fig. 3:**
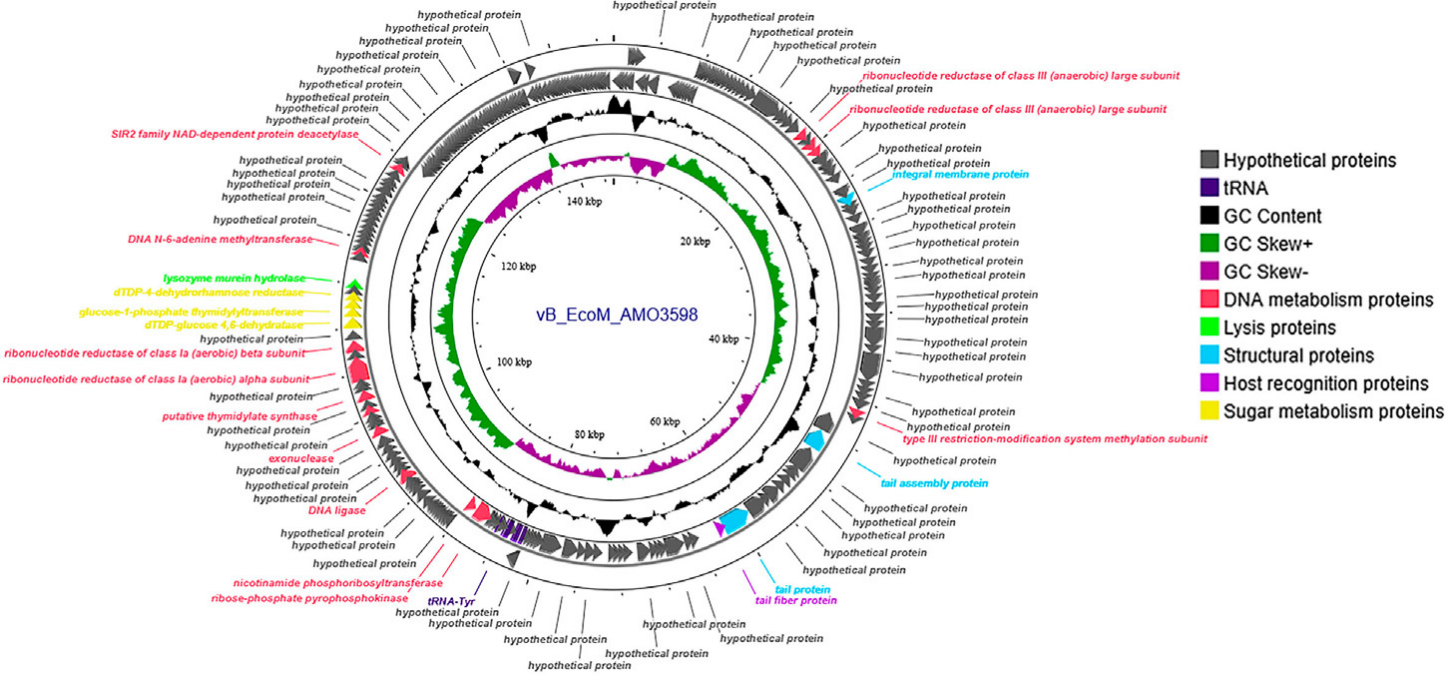
The genome map of the vB_EcoM_AMO3598 bacteriophage built using the CGView server (https://proksee.ca/ assessed on 10th April 2025).

Open reading frame (ORF) prediction using the standard genetic code identified 290 putative protein-coding genes, including 13 tRNAs. 24 predicted proteins had assigned functions and were categorized into groups: structural, lysis, DNA and sugar metabolism, and host recognition proteins (Fig. 3).

Analysis with PhageLeads reveals that there is no presence of virulent factor, antimicrobial resistance, or temperate lifecycle encoding genes in the genome of the vB_EcoM_AMO3598 bacteriophage.

**Fig. 4:**
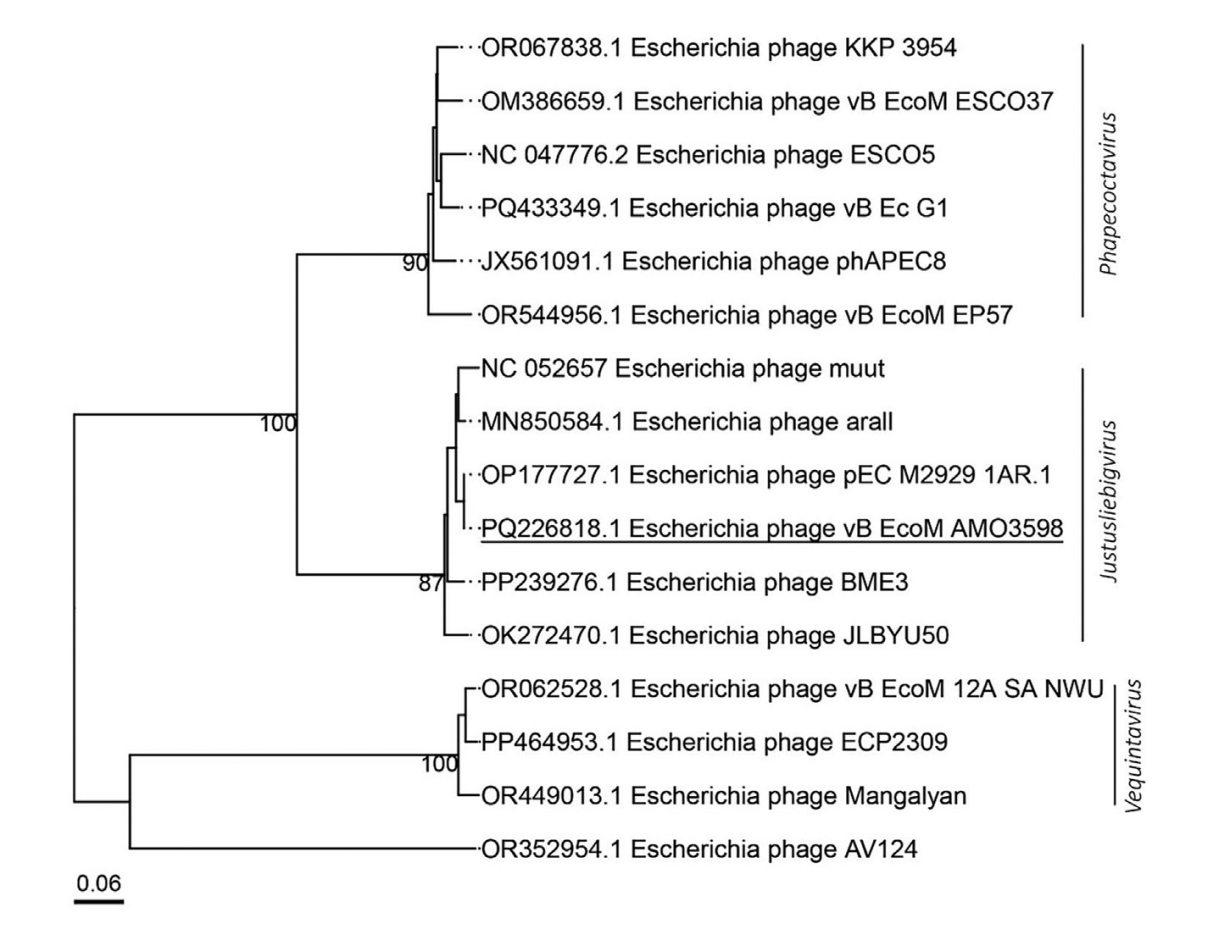
Phylogenetic tree generated by the VICTOR web service (https://victor.dsmz.de; accessed on 10 April 2025). All pairwise comparisons of the nucleotide sequences were conducted using the Genome-BLAST Distance Phylogeny (GBDP) method with the settings recommended for prokaryotic viruses.

The genomic similarity of vB_EcoM_AMO3598 with other representatives of the genus was analyzed using the VICTOR algorithm [6]. Phylogenetic analysis established that the studied bacteriophage belongs to the subfamily *Stephanstirmvirinae*, genus *Justusliebigvirus* (Fig.4).

## 4 Experimental Design, Materials and Methods

Clinical, antibiotic-resistant strains of *E. coli* were provided by the microbiological laboratory of the National Hospital of the Medical Center of the Presidential Administration of the Republic of Kazakhstan (Table 1).

The bacteria were cultured in TSB for 18–20 h at 37 °C on a shaker at 100 rpm.

Water samples were taken from the collector of the municipal sewage treatment plant at a depth of 1 −2 m into a sterile 500 ml bottle. The collected sample was transported in the dark at 4 - 8 °C. After delivery, the aqueous sample was filtered sequentially through a paper filter and a 0.45 µm bacterial filter. For phage enrichment, 1 mL of *E. coli* 3598 bacterial suspension and 1 mL of 10x TSB were added to 10 mL of the resulting filtrate and incubated at 37 °C for 16 h [7]. After the incubation, the bacterial suspension was centrifuged for 30 min at 4500 × *g* and filtered through a 0.45 µm bacterial filter.

The presence of lytic bacteriophages in the samples was confirmed and further isolation of their pure lines was performed by the double-layer agar method [8]. The enriched filtrate was titrated until a series of 10-fold dilutions was obtained. Each dilution was mixed with the exponential-phase culture of the corresponding host; then 3 ml of soft TSA agar (0.7 % w/v) was added, and the mixture was poured onto TSA (1.5 % w/v) plates. After solidifying, the plates were incubated for 16 h at 37 °C. After the incubation, the plates were observed for the presence of plaques. A single plaque from the plate with the maximum dilution was picked based on its size and clarity using a sterile pipette tip and was resuspended in a tube with 2 ml of TSB. The tube with the plaque was left at 4 °C for 4 h to allow the phage to diffuse into the TSB. Then, the tube was centrifuged at 4500 × *g* for 30 min, and the supernatant was filter-sterilized using a SPHEROS Syringe filter (PES, 0.45 µm). The purified phage was propagated using the *E. coli* 3598 host bacteria. This complete cycle was repeated at least 3 times until PFUs of only one morphotype were obtained [9].

The determination of the lytic activity spectrum of vB_EcoM_AMO3598 was carried out using the spot test technique. The bacterial lawns of all the selected bacterial hosts were prepared on TSA plates, then 100 µl of purified phage lysate of vB_EcoM_AMO3598 was applied to the edge of each Petri dish, and the formed drop was allowed to drip down to the opposite edge of the dish, and then they were incubated at 37 °C for 16 h. At the end of the incubation period, the formed lysis zones were visually evaluated. The absence of the lysis zone showed the inability of vB_EcoM_AMO3598 to lysis the corresponding bacterial strain, the presence of the lysis zone and its area allowed to assess the lytic activity of the tested bacteriophage in relation to the corresponding bacterial strain.

To determine the adsorption rate, 5 ml of the host bacterial suspension with a titer of 10^8^ CFU/ml was mixed with 5 ml of the phage-containing sample with a titer of 10^8^ PFU/ml. The resulting mixture was incubated at 37 °C, and 1 ml samples were taken during the incubation at 0, 2, 4, 6, 10, and 15 min after the start of the incubation. Each sample was centrifuged, and the amount of non-adsorbed bacteriophages in the resulting supernatant was determined using the agar layer method [9].

For a one-step growth assay, the phage-containing sample was mixed with 1 ml of the host bacterial culture at an MOI of 0.1. The resulting mixture was incubated at 37 °C for 6 min, following by centrifugation at 5000 g for 10 min to remove unadsorbed bacteriophages. The resulting pellet was resuspended in 10 mL of TSB and incubated at 37 °C. At 10-minute intervals for 60 min, 1 mL of the sample was withdrawn to determine the titer of viral particles using the agar layer method. A plaque assay was performed to determine the titer of viral particles. Based on the obtained titer values, a growth curve was constructed and the latent period and growth burst were determined. For statistical reliability, all experiments were performed 3 times, the values were expressed as the mean with a standard deviation [9].

The structure of the isolated bacteriophage was studied by transmission electron microscopy (TEM) at an instrumental magnification of 60–120 thousand times and a voltage of 80 kV using the HT7800 (Hitachi, Japan) microscope [10].

The DNA extraction was performed with a PureLink viral DNA/RNA mini kit (Thermo Fisher Scientific, USA). The purity and concentration of the isolated DNA were assessed using a NanoQuant plate on a multifunctional reader Infinite 200 Pro (Tecan, Switzerland) and a Qubit 3.0 fluorometer (Thermo Fisher Scientific, USA), respectively. For library preparation, 1 ng of genomic DNA was used with the Nextera XT Library Preparation Kit. The fragment size distribution of the resulting library was evaluated using an Agilent 2100 Bioanalyzer (Agilent, USA) in combination with the Agilent High Sensitivity DNA Kit. Sequencing was performed on the Illumina MiSeq platform, generating paired-end reads with an average length of 300 base pairs (Illumina kit v3). The sequence read quality was evaluated using FastQC (Galaxy Version 0.74) [11] and trimmed with Trimmomatic (Galaxy Version 0.38.1) [12]. The phage genome was assembled using SPAdes 3.12.0 [13]. Predicted coding sequences (CDS) were annotated using PROKKA 1.7 [14] and manually edited. The coding regions of tRNAs were identified using tRNAscan-SE [15]. Phage genome termini and genome completeness were determined by CheckV version 0.8.1 from PhageScope [16]. PhageLeads together with the integrated ABRicate tool were used to check for the absence of lysogeny factors, antimicrobial resistance (AMR) and virulence genes [17]. The phage taxonomy was determined on the basis of phylogenetic analysis using sequences of closely related genomes in the GenBank database. A phylogenetic tree was constructed with the VICTOR web service using the method of genomic phylogeny and classification of prokaryotic viruses. All pairwise comparisons of nucleotide sequences were performed using the Genome-BLAST Distance Phylogeny (GBDP) method according to the settings recommended for prokaryotic viruses [18]. All the tools were run with default parameters.

The raw genome sequencing data of Illumina MiSeq were submitted to NCBI SRA database in FASTQ format: SRS24680452, with BioSample: SAMN47875066, under BioProject PRJNA1249015. The assembled genome is available in the NCBI GeneBank under PQ226818.1 [19].

## Limitations

Despite the encouraging results obtained, confirming the therapeutic potential of the studied bacteriophage against *E. coli*, there are minor limitations: the selection and diversity of *E. coli* strains used to assess the host range were based on the availability and clinical relevance, rather than being a fully representative sample; further studies with a more genetically and geographically diverse collection of *E. coli* will allow us to determine the spectrum of phage activity more accurately. Nevertheless, the phage exhibited pronounced lytic activity against several clinically significant strains, and its genomic and phenotypic characteristics confirm its potential for use in targeted phage therapy. In the future, it is planned to expand the spectrum of tested strains and study possible synergistic effects with other phages or antimicrobial agents.

## Ethics Statement

Authors have read and follow the ethical requirements for publication in Data in Brief and confirming that the current work does not involve human subjects, animal experiments, or any data collected from social media platforms.

## CRediT Author Statement

**Madina Alexyuk:** Conceptualization, Methodology, Formal analysis, Validation, Investigation, Resources, Writing - Original Draft, Writing - Review & Editing, Funding acquisition. **Andrey Bogoyavlenskiy:** Formal analysis, Data Curation, Writing - Review & Editing. **Kuralay Akanova:** Investigation, Visualization. **Yergali Moldakhanov:** Investigation, Visualization. **Adolat Manakbayeva:** Formal analysis, Data Curation. **Timur Kerimov:** Formal analysis, Data Curation. **Vladimir Berezin:** Supervision, Writing - Review & Editing. **Pavel Alexyuk:** Conceptualization, Methodology, Validation, Investigation, Writing - Original Draft, Writing - Review & Editing, Project administration.

## Data Availability

NCBI/GeneBankEscherichia phage vB_EcoM_AMO3598, complete genome (Original data).
